# The Atypical Receptor CCRL2 (C-C Chemokine Receptor-Like 2) Does Not Act As a Decoy Receptor in Endothelial Cells

**DOI:** 10.3389/fimmu.2017.01233

**Published:** 2017-10-06

**Authors:** Chiara Mazzotti, Vincenzo Gagliostro, Daniela Bosisio, Annalisa Del Prete, Laura Tiberio, Marcus Thelen, Silvano Sozzani

**Affiliations:** ^1^Laboratory of Experimental Immunology, Department of Molecular and Translational Medicine, University of Brescia, Brescia, Italy; ^2^Humanitas Clinical and Research Centre, Rozzano, Italy; ^3^Institute for Research in Biomedicine, Università della Svizzera italiana, Bellinzona, Switzerland

**Keywords:** chemokine, endocytosis, G protein-coupled receptor, intracellular trafficking, scavenger receptor, atypical chemokine receptor, chemerin

## Abstract

C-C chemokine receptor-like 2 (CCRL2) is a non-signaling seven-transmembrane domain (7-TMD) receptor related to the atypical chemokine receptor (ACKR) family. ACKRs bind chemokines but do not activate G protein-dependent signaling or cell functions. ACKRs were shown to regulate immune functions *in vivo* by their ability to scavenge chemokines from the local environment. This study was performed to investigate whether CCRL2 shares two of the main characteristics of ACKRs, namely the ability to internalize and scavenge the ligands. Cell membrane analysis of CCRL2-transfected cells revealed a weak, constitutive, ligand-independent internalization, and recycling of CCRL2, with a kinetics that was slower than those observed with ACKR3, a prototypic ACKR, or other chemotactic signaling receptors [i.e., chemokine-like receptor 1 and C-X-C motif chemokine receptor 2]. Intracellularly, CCRL2 colocalized with early endosome antigen 1-positive and Rab5-positive vesicles and with recycling compartments mainly characterized by Rab11-positive vesicles. CCRL2-transfected cells and activated mouse blood endothelial cells, that endogenously express CCRL2, were used to investigate the scavenging ability of CCRL2. These experiments confirmed the ability of CCRL2 to bind chemerin, the only recognized ligand, but excluded the ability of CCRL2 to perform scavenging. Collectively, these results identify unique functional properties for this member of the non-signaling 7-TMD receptor family.

## Introduction

Chemokines are soluble mediators that regulate immune functions and development, mainly through their ability to promote leukocyte trafficking. Chemokines bind seven-transmembrane domain (7-TMD) receptors. Chemokine receptors can be divided in two main functional groups: the “classical” G protein-coupled signaling receptors (GPCR) and the atypical chemokine receptors (ACKRs) (1). ACKRs do not signal through G proteins, but rather they promote ligand internalization and degradation. In some cases, ACKRs can also transport the ligand across the cell (2). By these mechanisms, ACKRs were shown to regulate chemokine availability, shape the chemokine gradient and regulate inflammatory responses *in vivo* (2–5). To date, four 7-TMD receptors have been included into the ACKR nomenclature (ACKR1–4). An additional 7-TMD receptor, called C-C chemokine receptor like 2 (CCRL2), was proposed as the fifth member of the ACKR family, pending confirmation of its ability to bind chemokines (1).

Similar to ACKRs, CCRL2 presents in the cytoplasmic extension of the third transmembrane helix an altered amino acidic sequence (QGYRVFS) in place of the conserved motif DRYLAIV, which is required for the triggering of G protein-mediated responses (1). Although an earlier study reported that CCRL2 can signal through MAPK activation and promote cell migration (6), a recent report clearly excluded ERK1/2 activation by CCRL2 (7). Thus, CCRL2 does not trigger classic G protein-mediated signaling or mediate cell migration.

Mouse CCRL2 maps within the C-C chemokine receptors gene cluster on chromosome 9 and shows high homology with other C-C chemokine receptors, sharing 35.8% amino acidic identity with C-C motif chemokine receptor 5 (CCR5) and 34.9% with C-C motif chemokine receptor 1 (CCR1) (8). Mouse CCRL2 is expressed by several leukocytes, including dendritic cells, macrophages, neutrophils, and microglia and is rapidly upregulated following stimulation with proinflammatory stimuli, such as LPS and TNF-α (9–14). CCRL2 is also expressed by barrier cells, such as lymphatic and blood endothelial cells (15, 16) and bronchial and intestinal epithelial cells (17, 18). CCRL2 was shown to play a role in the regulation of immunity. Indeed, CCRL2-deficient mice were protected in a model of IgE-induced anaphylaxis (19), in a model of ovalbumin-induced lung hypersensitivity (13) and in experimental models of inflammatory arthritis (10). On the contrary, CCRL2-deficient mice developed an exacerbated inflammatory response when used in a model of experimental autoimmune encephalomyelitis (20).

In the past years, it was proposed that CCRL2 may act as the receptor for some chemokines, such as CCL2, CCL5, CCL7, CCL8, CCL19, and CCL21 (6, 18, 21–23), but these results have been questioned by other groups (1). Instead, CCRL2 was described to bind the non-chemokine chemotactic factor chemerin (7, 16, 19, 24). In the currently accepted model, CCRL2 binds chemerin at the N-terminus and leaves the C-terminus accessible for the interaction with cells expressing chemokine-like receptor 1 (CMKLR1), the functional chemerin receptor. Therefore, CCRL2 is proposed to act as a chemerin presenting molecule at the surface of barrier cells (19, 25). This model is supported by *in vitro* and *in vivo* results (15, 16) and assumes that CCRL2, differently from all the other ACKRs, does not internalize, recycle and scavenge the ligand. Nevertheless, human CCRL2 was proposed to undergo constitutive internalization of a putative ligand (CCL19) in the pre-B cell line Nalm6 and in CHO-K1-transfected cells (22, 23). Others did not confirm such internalization (7, 16, 19). Thus, the scavenging and recycling properties of CCRL2, as well as its biological function, are still a matter of debate.

This study was performed to investigate the internalization, recycling and scavenging properties of CCRL2, ectopically expressed in cell lines or naturally expressed by mouse blood endothelial cells. The results here reported formally demonstrate that CCRL2, differently from all the other ACKRs, is devoid of ligand scavenging properties, and support its proposed function as a chemerin anchoring protein on the surface of endothelial cells.

## Materials and Methods

### Cell Culture and Transfection

COS-7 cells and HeLa cells were cultured in DMEM supplemented with 10% fetal bovine serum, 100 U/ml penicillin/streptomycin, and 2 mM glutamine (all from Gibco by Life Technologies, Carlsbad, CA, USA). For transient transfections, cells were seeded on glass slides coated with poly-l-lysine in order to obtain 90% of confluency on the day of transfection. After 24 h, transfection was performed using Lipofectamine 2000 (ThermoFisher Scientific, Waltham, MA, USA) according to manufacturer’s protocol. 1G11 cells are cultures of primary mouse blood endothelial cells, used between passage 15–20. In culture, 1G11 cells retain the normal expression of CD31, ICAM1, VCAM1, and E-selectin up to passage 40 (15, 26). When indicated, 1G11 cells were treated either with complete medium or with IFNγ (50 ng/ml), TNFα (20 ng/ml), and LPS (1 µg/ml) in complete medium overnight. After 18 h, cells were rinsed with PBS before performing experiments. For stable transfection, cells were transfected with Lipofectamine 2000 as previously described. After 24 h, cells were passaged to reach a subconfluent concentration. They were maintained and passaged for 2 weeks in the presence of 1 mg/ml Geneticin (Gibco by Life Technologies). After 2 weeks, cells were detached and sorted for receptor expression as described in Section “Flow Cytometry and Cell Sorting.”

### Plasmids

C-C chemokine receptor-like 2, CMKLR1, and C-X-C motif chemokine receptor 2 (CXCR2) were amplified by PCR, cut with EcoRI and XbaI restriction enzymes and cloned into pCDNA3.1 plasmids downstream the acyl carrier protein (ACP) tag (FWWGLDSLDTVELVMA) or the peptidyl carrier protein (PCP) tag (GDSLSWLLRLLN), preceded by the leader sequence (MRLCIPQVLLALFLSMLTGPGEGSRRRATQ) (27, 28).The following primers were used: mCCRL2 (5′-GAATTCGATAACTACACAGTGGCCCCG-3′ and 5′-TCTAGATTATATTATATCCTG-3′), mCMKLR1 (5′-ATTGAATTCGATGAGTACGACGCTTAC-3′ and 5′-ATTTCTAGATCAGAGGGTACTGGTCTCC-3′), and mCXCR2 (5′-ATAATAGAATTCGGAGAGTTCAAGGTGGATAAGTTC-3′ and 5′-ATAATATCTAGATTAGAGGGTAGTAGAGGTGTTTG-3′). ACKR3 was cloned downstream the PCP tag as previously described (27).

Non-tagged mCCRL2 and mCXCR2 were cloned in pCDNA3.1 using the following forward primers: mCCRL2 (5′-ATTGGAATTCGAATGGATAACTACACAGTGGC-3′) and mCXCR2 (5′-ACCAAGCTTATGGGAGAATTCAAG-3′).

Two truncated forms of mCCRL2 were cloned downstream the tag, the first ending at amino acid 310A before the helix-VIII (H8^−^), the second at amino acid 319F after helix-VIII (H8^+^). The truncated forms were cloned using the following reverse primers: H8^−^ (5′-ATTTCTAGACTAGGCCTTCCGGTCAAGAAGC-3′) and H8^+^ (5′-ATTTCTAGACTAGAACAGGCTGCGAAGGTATCTCAT-3′).

### Enzymatic Labeling and Quantification of Receptor Internalization

Two short peptides, derived from the PCP and the ACP, act as substrates for the phosphopantetheinyl transferases (PPTases) Sfp and AcpS. Therefore, PPTases can label receptors tagged with either the ACP or PCP tag, in the presence of CoA functionalized with a fluorophore, as described (29, 30). Cells were seeded on glass slides and transiently transfected with the plasmids described above. After 24 h, receptors were labeled using either the enzyme AcpS (ACP tag) or Sfp (both ACP and PCP tags) that transfer the fluorophore from the CoA to the tag. Cells were incubated at 17°C during the reaction, to prevent receptor endocytosis, and then washed thoroughly with PBS at room temperature. Next, cells were incubated at 37°C in complete medium in the presence of the different stimuli for the indicated times. CoA functionalized with 594 fluorophore (CoA-594) was prepared as described (27, 31); Sfp and AcpS were from New England BioLabs. When required, before fixation the membrane was marked by incubating cells with Wheat Germ Agglutinin (WGA), Alexa Fluor^®^ 488 conjugate (Invitrogen, ThermoFisher Scientific, Waltham, MA, USA), diluted 1:150 in HBSS buffer, at 17°C for 10 min. Fluorescence microscopy of living or fixed cells was performed with Zeiss Observer.Z1 microscope using a 100× (1.4 numerical aperture) oil immersion lens and Apotome2 for optical sectioning. Images were acquired using AxioVision software. Images were analyzed using ImageJ 1.48 (National Institutes of Health; http://rsb.info.nih.gov/ij/) to quantify receptor internalization. Two gates were designed for each cell with the help of the WGA staining: one including the membrane and one excluding the membrane. After converting images to 8-bit, a threshold of fluorescence was defined and the number of pixels over the threshold was counted inside the gates. Receptor internalization is expressed as ratio between internal and total fluorescence measured for each single cell.

### Immunofluorescence

Cells were enzymatically labeled, fixed in 2% paraformaldehyde, 7% sucrose, in PBS. Free aldehydic groups from paraformaldehyde were covered incubating with 50 mM NH_4_Cl for 10 min. Buffer containing 0.25% Saponin, 5% normal goat serum, and 2% serum bovine albumine in PBS was used for permeabilization and blocking; incubation with antibodies was performed in the presence of 0.25% Saponin. The following primary antibodies were used: mouse antiearly endosome antigen 1 (anti-EEA1) 1:100 (610456, BD Biosciences, San Jose, CA, USA); rabbit antilysosomal-associated membrane protein 1 (anti-LAMP1) 1:500 (ab24170; abcam, Cambridge, UK); rabbit anti-Rab4 1:100 (sc-312, Santa Cruz Biotechnology, Santa Cruz, CA, USA); rabbit anti-Rab5 1:100 (sc-28570, Santa Cruz Biotechnology); rabbit anti-Rab7 1:100 (sc-10767, Santa Cruz Biotechnology); rabbit anti-Rab11 10 µg/ml (71-5300, Invitrogen); mouse antivesicle-associated membrane protein 2 (anti-VAMP2) 1:500 (Synaptic Systems, Gottingen, Germany); rabbit anti-VAMP3 1:200 (ThermoFisher Scientific); rabbit anti-Syntaxin-6 1:100 (Synaptic Systems); and mouse antitransferrin receptor (anti-TfR) 1:200 (Invitrogen). The secondary antibodies used were: goat anti-Mouse Alexa Fluor^®^ 405 conjugate 1:250 (Invitrogen); goat anti-Rabbit DyLight 405 conjugate 1:500 (ThermoFisher Scientific). Immunofluorescence microscopy was performed as described before.

### Flow Cytometry and Cell Sorting

Hela cells transiently transfected with the truncated forms of CCRL2 were detached and labeled using AcpS in suspension at 17°C. Cells were then washed and analyzed by flow cytometry. Alternatively, CCRL2 was labeled using anti-mCCRL2-PE (clone BZ2E3, BD Pharmigen, BD Biosciences). CMKLR1 was labeled using anti-mCMKLR1-PE (Clone # 477806, R&D Systems, Minneapolis, MN, USA). Flow cytometry analysis was performed using MACSQuant^®^ Analyzer Miltenyi, files were plotted using FlowJo software (TreeStar Inc.).

For sorting, cells were labeled with appropriate antibodies, resuspended in PBS containing 0.5% BSA and 5 mM EDTA, filtered through a cell strainer and sorted with FACSAria III cell sorter (BD).

### Enzyme-Linked Immunosorbent Assay (ELISA)

Cells were incubated in DMEM, containing 4 mM HEPES and 1% BSA, in the presence or in the absence of 1 nM of chemerin (R&D) or CCL19 (Peprotech, Rocky Hill, NJ, USA) for the indicated times. Where indicated, cells were preincubated with 80 µm Dynasore (dynamin inhibitor, Sigma, St. Louis, MO, USA). After incubation, residual ligand in the supernatant was quantified by ELISA (DY2324 and DY361, R&D).

To evaluate chemerin into the cells, cells were incubated as previously described, in the absence or in the presence of 1 nM chemerin at 37°C for 2 h. Then plates were put in ice and rinsed with PBS. Cells were washed with different buffers for 5 min at room temperature: PBS, acidic buffer [150 mM NaCl, 100 mM glycine, pH = 3, in water (32)], or acidic followed by high salt buffer (2 M NaCl in water). After rinsing with PBS, cells were lysed for 30 min in ice (1% Triton X-100, 5 mM EDTA, in PBS), then centrifuged (16,000*g*) at 4°C for 15 min. Internalized chemerin was evaluated by ELISA from supernatants obtained after centrifugation. The quantity of chemerin was normalized over total protein content, quantified using Bradford reagent (Biorad, Hercules, CA, USA).

### Gene Expression

Cells were lysed using TRIzol (Life Technologies) and total RNA was extracted following manufacturer’s protocol. cDNA was prepared from 500 ng of total RNA using M-MLV Reverse Transcriptase (ThermoFisher Scientific). mRNA expression was determined by quantitative PCR using SYBR Green (BioRad). PCR reactions were performed using StepOnePlus Real-Time PCR system (Applied Biotechnologies by ThermoFisher). Threshold cycle values (Ct) were determined and relative mRNA expression was analyzed by the comparative ΔCt method. Data were normalized to the mRNA expression of RPL32 (murine samples) or GAPDH (human samples). The following primers were used: mCCRL2 (5′-TGTGTTTCCTGCTTCCCCTG-3′ and 5′-CGAGGAGTGGAGTCCGACAA-3′), mCMKLR1 (5′-CCATGTGCAAGATCAGCAAC-3′ and 5′-GCAGGAAGACGCTGGTGTA-3′), mRPL32 (5′-GCTGCCATCTGTTTTACGG-3′ and 5′-TGACTGGTGCCTGATGAACT-3′), and hGAPDH (5′-AGCCACATCGCTCAGACA-3′ and 5′-GCCCAATACGACCAAATCC-3′).

### Statistical Analysis

When not specified, graphics represent mean values ± SEM. Statistical significance between the experimental groups was determined by using one- or two-way ANOVA with Bonferroni posttest, or unpaired *t*-test (GraphPad Prism; GraphPad Software, La Jolla, CA, USA). *p*-Values of <0.05 were considered significant.

## Results

### CCRL2 Displays a Slow, Constitutive, Ligand-Independent Internalization

**Figure 1:**
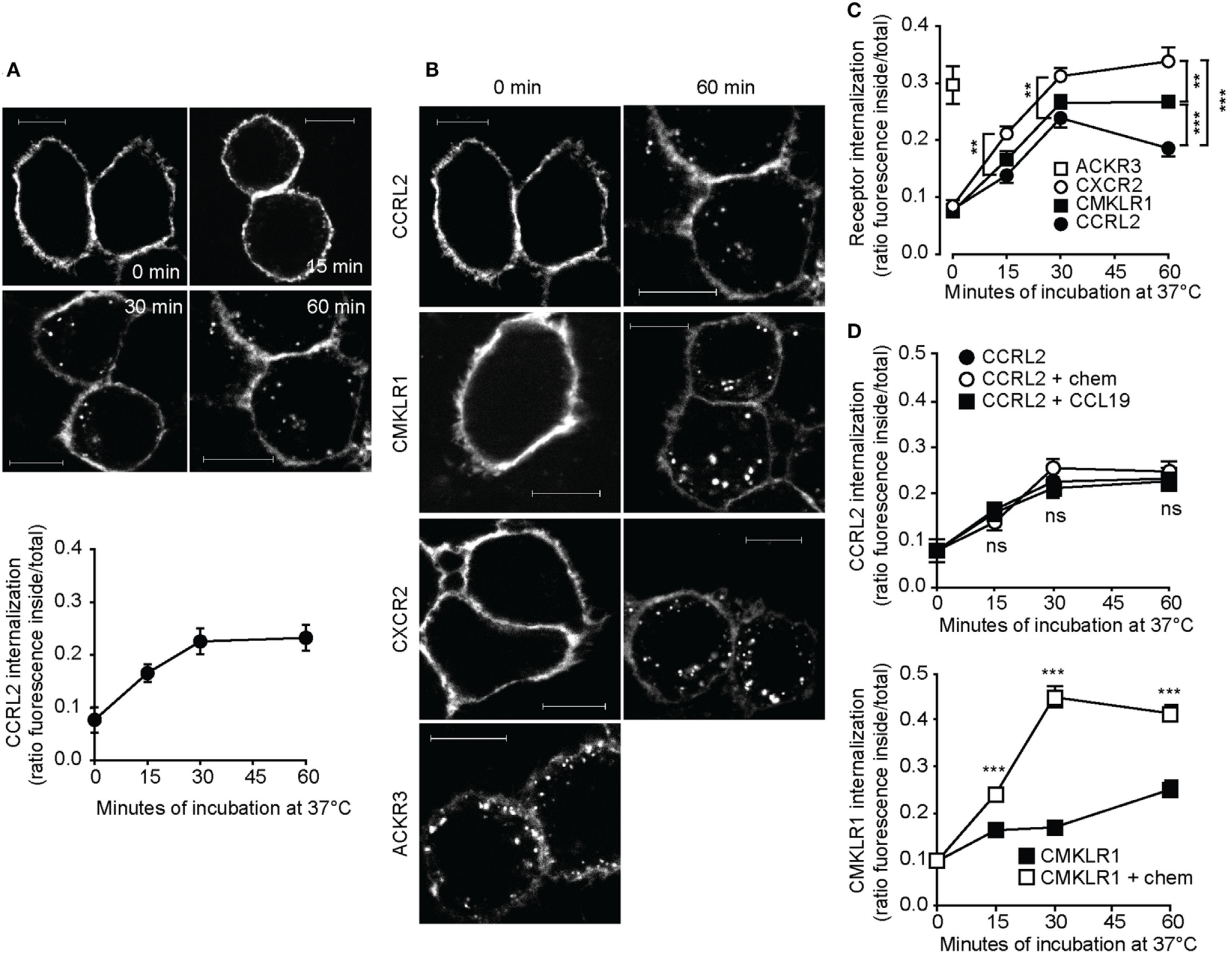
C-C chemokine receptor-like 2 (CCRL2) internalization is ligand independent. HeLa cells were transfected with acyl carrier protein (ACP)-CCRL2, ACP chemokine like receptor 1 (CMKLR1), peptidyl carrier protein-C-X-C motif chemokine receptor 2 (PCP-CXCR2), or PCP-atypical chemokine receptor 3 (ACKR3) expression vectors and receptors were labeled. Cells were then incubated at 37°C in serum-free medium for the indicated times and fixed. Images were taken using 100× magnification and analyzed using ImageJ 1.48 (NIH). **(A)** Representative images (above) and quantification (below) of ACP-CCRL2 internalization after 0, 15, 30, 60 min of incubation. Internalization is expressed as the ratio between internal and total fluorescence, calculated on each single cell. One experiment representative of two is shown; each point represents the average value ± SEM calculated on 19–32 cells. **(B)** Representative images of labeled ACP-CCRL2, ACP-CMKLR1, PCP-CXCR2, and PCP-ACKR3, at 0 and 60 min incubation. Scale bars: 10 µm. **(C)** ACP-CCRL2, ACP-CMKLR1, PCP-CXCR2, and PCP-ACKR3 internalization, calculated as in **(A)**. One experiment representative of two is shown; each point represents the average value ± SEM calculated on 20–50 cells. **p < 0.01 and ***p < 0.001 by two-way ANOVA. **(D)** (Above) After labeling of ACP-CCRL2, cells were incubated in the presence of 100 nM CCL19 or 100 nM chemerin for the indicated times. Internalization was measured as described before; analysis by two-way ANOVA; ns, not significant (CCRL2 vs. CCRL2 + chem and CCRL2 + CCL19). (Below) After labeling of ACP-CMKLR1, cells were incubated in the presence or in the absence of 100 nM chemerin for the indicated times; internalization was measured as described before. ***p < 0.001 by two-way ANOVA (CMKLR1 vs. CMKLR1 + chem). In both the graphs, one representative of two independent experiments is shown, each point represents the average values ± SEM calculated on 20–50 cells.

To study the intracellular trafficking, acyl carrier protein (ACP)-tagged CCRL2 was expressed in HeLa cells and receptors were enzymatically labeled using a fluorescent dye. Living cells are impermeable to the dye, therefore this reaction, when performed before fixation, labels only the tagged proteins present at the cell surface. Indeed, immediately after labeling the fluorescence signal was detected only at the plasma membrane level (Figure 1A). After a few minutes of incubation at 37°C, fluorescent vesicles started to be detectable also in the cytoplasm and progressively accumulated in the perinuclear region (Figure 1A). The degree of internalized fluorescence increased over time reaching about 20% of total fluorescence after 60 min incubation (Figure 1A). Since ACKRs are characterized by rapid constitutive internalization (33–35), the kinetics of CCRL2 internalization was compared to that of ACKR3, a receptor belonging to the ACKR family (34), and to that of two chemotactic receptors, namely CXCR2 and CMKLR1. Tagged CCRL2, CXCR2, CMKLR1, and ACKR3 were expressed in HeLa cells and enzymatically labeled. All the receptors showed comparable levels of baseline internalization, with the only exception of ACKR3. Indeed, intracellular ACKR3-positive vesicles were clearly detectable at time zero, indicating that receptor internalization occurred even during the washing steps performed at room temperature (Figures 1B,C). The kinetics of CCRL2 internalization was similar to those of CXCR2 and CMKLR1, although the degree of internalization of CCRL2 was lower (Figure 1C). A similar pattern of internalization was observed when CCRL2 was expressed in COS-7 cells (data not shown).

Since 7-TMD receptor internalization is usually increased upon ligand binding interaction (36), the effect of known or putative ligands on CCRL2 and CMKLR1 internalization was investigated. The addition of chemerin or CCL19 did not modify the internalization kinetics of CCRL2 (Figure 1D, upper panel). Instead, CMKLR1 internalization was rapidly increased upon chemerin stimulation (Figure 1D, lower panel). A panel of chemokines was tested to further explore a possible ligand-induced internalization of CCRL2. Of 29 chemokines tested (Table 1), none of them enhanced the degree of CCRL2 internalization (not shown).

**Table 1:** Chemokines that do not induce CCRL2 internalization.

**CC**	CCL2	CCL3	CCL4	CCL5	CCL7	CCL8	CCL13	CCL17
	CCL18	CCL20	CCL22	CCL23	CCL24	CCL25	CCL26	
**CXC**	CXCL1	CXCL2	CXCL4	CXCL5	CXCL6	CXCL7	CXCL8	CXCL10
	CXCL11	CXCL12	CXCL13	CXCL14				
**CX3C**	CX3CL1							
**XCL**	XCL1							

*Hela cells transfected with ACP-CCRL2 and enzymatically labeled were incubated with 100 nM of the chemokines listed subsequently*.

### Internalized CCRL2 Follows the Slow Recycling Pathway

**Figure 2:**
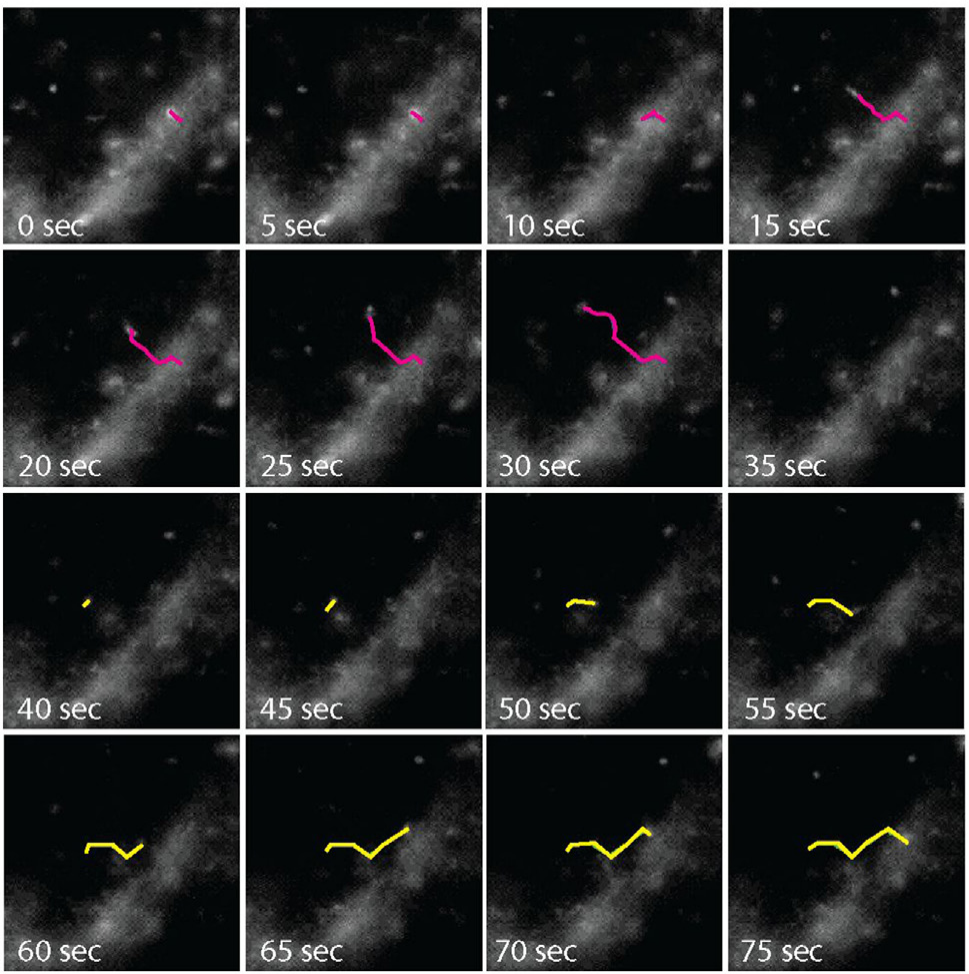
Time-lapse microscopy analysis of C-C chemokine receptor-like 2 (CCRL2)-positive vesicles. COS-7 cells expressing acyl carrier protein (ACP)-CCRL2 were enzymatically labeled. After labeling, cells were placed under the microscope at 37°C, in the presence of 5% CO_2_, and observed by time-lapse microscopy every 5 s. Pink line, endocytosing vesicle; yellow line, exocytosing vesicle.

**Figure 3:**
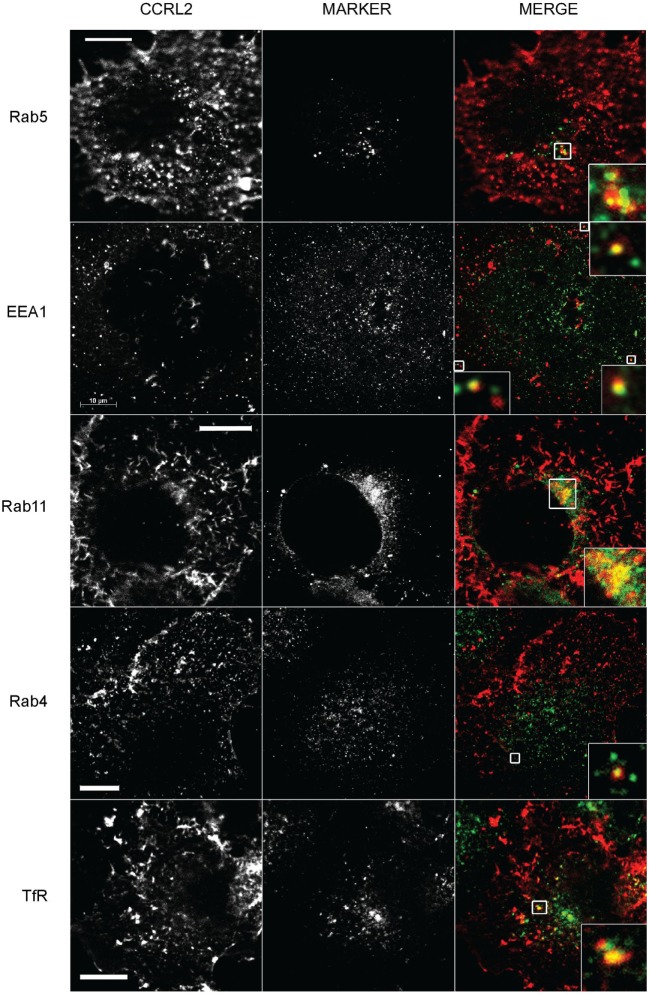
C-C chemokine receptor-like 2 (CCRL2)-positive vesicles colocalize with markers of the slow recycling pathway. COS-7 cells expressing acyl carrier protein (ACP)-CCRL2 were enzymatically labeled. After labeling, cells were incubated at 37°C for 20 min, fixed, permeabilized and mounted after staining for the indicated markers. Images were taken at 100× magnification. Labeled CCRL2 and the fluorescent intracellular markers are shown in the first and the second columns, respectively. The third column represents the merging of CCRL2 (red) and marker (green) fluorescences. Inserts represent magnifications of the boxed areas. Scale bars: 10 µm.

Time lapse imaging of membrane ACP-CCRL2-labeled cells showed that CCRL2-positive vesicles moved from the cell membrane toward the cytoplasm and back to the cell membrane (Figure 2; Video S1 in Supplementary Material). This observation suggested that, upon internalization, CCRL2 could recycle to the cell membrane. To prove this hypothesis, the colocalization of CCRL2 with specific markers of distinct intracellular compartments was investigated by immunofluorescence. CCRL2 was found to colocalize with Rab5- and EEA1-positive early endosomes (Figure 3). From early endosomes, receptors can recycle to the plasma membrane either *via* Rab4^+^ vesicles (rapid recycling pathway) or alternatively *via* Rab11^+^ vesicles (slow recycling pathway) (37, 38). CCRL2 was mostly observed colocalized with Rab11^+^ and only occasionally with Rab4^+^ vesicles. Indeed, CCRL2 colocalized also with the TfR, a reference receptor for recycling pathways (Figure 3) (39). Of note, a significant portion of the internalized receptors was not associated with any of the endosomal markers. This is probably due to technical issues dealing with the different intensity of florescent staining of tagged receptors vs. moAb-labeled markers and with the flat shape of COS-7 cells that brings in close contact the upper and lower portion of the cell membrane. From EEA1-positive vesicles receptors can either recycle or be directed to lysosomal degradation. However, the lack of colocalization with Rab7 (a late endosome marker) or LAMP1 (a lysosome marker) argued against the possible localization of CCRL2 in lysosomal compartments. Similarly, the absence of colocalization with VAMP2 and VAMP3 and sintaxin-6 immunostaining excluded the association of CCRL2 with the Golgi compartment (Figure S1 in Supplementary Material).

### CCRL2 C-Terminus Is Dispensable for Receptor Internalization

**Figure 4:**
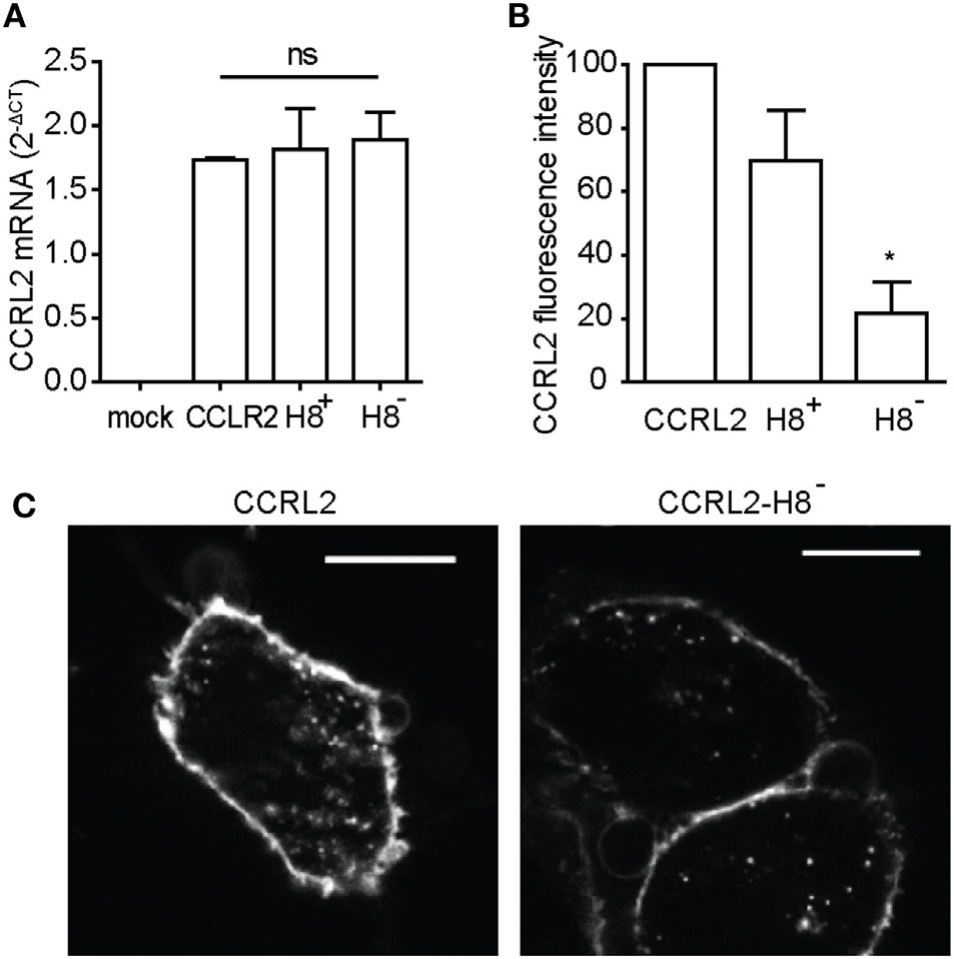
C-C chemokine receptor-like 2 (CCRL2) C-terminus is dispensable for receptor internalization. HeLa cells were transfected with a acyl carrier protein (ACP)-CCRL2, or with the truncated forms of ACP-CCRL2 (H8^+^ or H8^−^). **(A)** Total RNA was extracted 24 h after transfection and CCRL2 mRNA expression quantified by qPCR. 2^−ΔCT^ values are reported as average values of two independent experiments performed in triplicate. **(B)** 24 h after transfection, cell membrane receptors were fluorescently labeled and cells were analyzed by flow cytometry. Data were analyzed using the FlowJo software. The mean values of three independent experiments are shown; median fluorescence intensity of untransfected cells was subtracted and values were normalized over the mean fluorescence intensity of cells expressing non-truncated CCRL2 set at 100. **(C)** 24 h after transfection, cell membrane ACP-CCRL2 were fluorescently labeled. Cells were then placed at 37°C for 20 min and then fixed. Images were taken using 100× magnification. *p < 0.05 (H8^−^ vs. CCRL2) by Student’s *t*-test.

To investigate the mechanisms involved in CCRL2 internalization, two C-terminus truncated forms of the receptor were expressed in HeLa cells. One form, named CCRL2-H8^-^, is truncated after 310 A and lacks the entire C-terminus. The second form of the receptor, named CCRL2-H8^+^, is truncated after the amino acid 319 F and includes a structure called helix-VIII (H8). H8 is an alpha-helical structure present in class-A GPCRs and related to several molecular events, such as receptor expression, internalization, G protein coupling, regulation of activation and β-arrestin recruitment (40, 41). Figure 4B shows that, despite similar mRNA expression levels (Figure 4A), cell membrane expression of CCRL2-H8^−^ was reduced when compared to CCRL2-H8^+^ or the full-length CCRL2. These results suggest that the presence of H8 is required for the trafficking of CCRL2 from intracellular compartments to the cell membrane after its translation. Conversely, H8 seems to be dispensable for internalization, since the absence of H8 did not abrogate CCRL2 localization into intracellular vesicles (Figure 4C).

### CCRL2-Transfected Cells Internalize Chemerin

**Figure 5:**
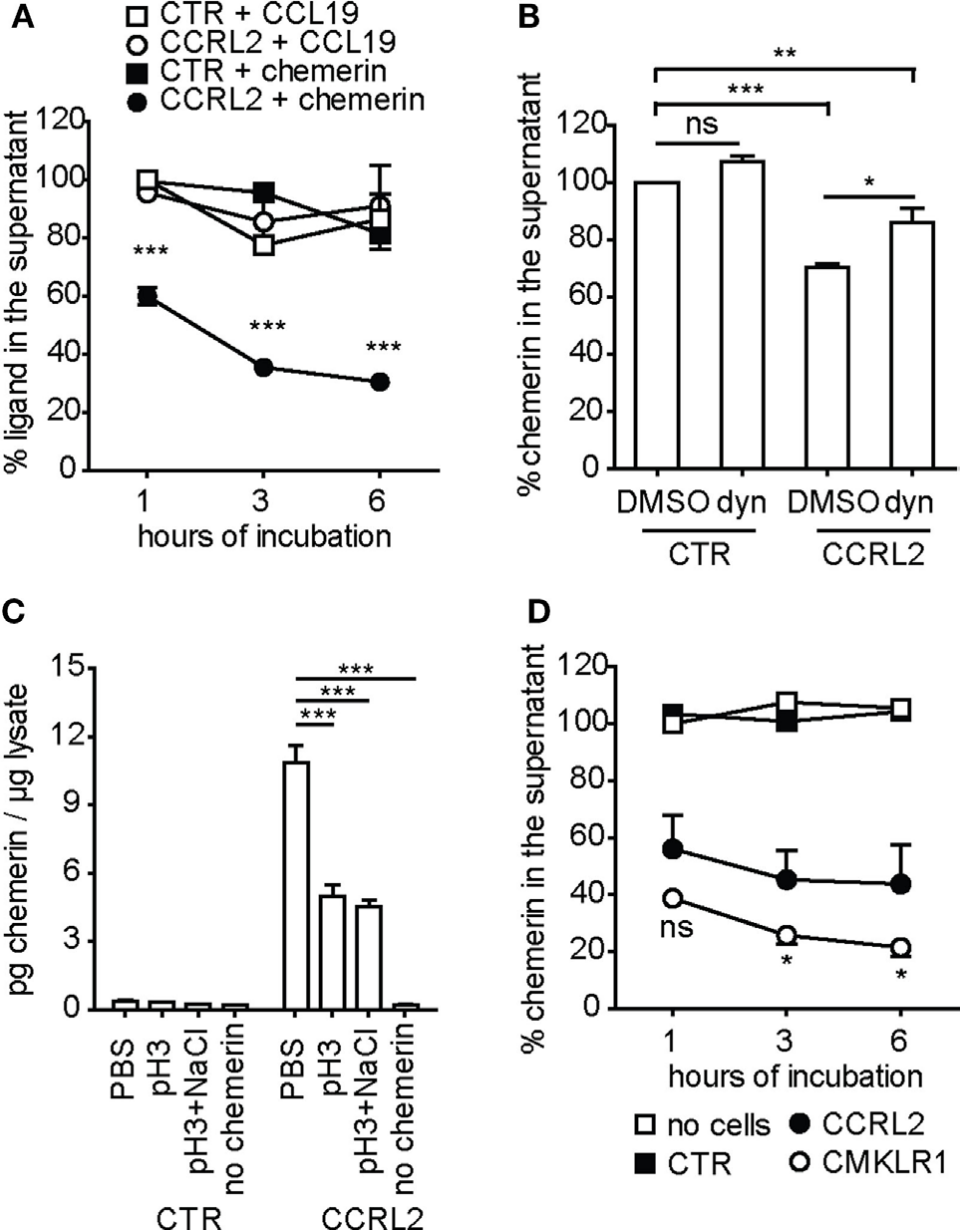
Chemerin, but not CCL19, is internalized by C-C chemokine receptor-like 2 (CCRL2)-transfected HeLa cells. **(A)** Hela cells stably expressing CCRL2 (CCRL2) or control cells (CTR) were incubated with 1 nM chemerin or 1 nM CCL19. At the indicated times, supernatants were collected and ligand concentration was assessed by enzyme-linked immunosorbent assay (ELISA). The mean values of three independent experiments are shown; the values for each ligand were normalized over the value of the respective control at 1 h of incubation. ***p < 0.001 (CCRL2 + chemerin vs. CTR + chemerin) by two-way ANOVA. **(B)** Hela cells stably expressing CCRL2 (CCRL2) or control cells (CTR) were pre-incubated with 80 µM Dynasore or 0.1% DMSO for 1 h, then chemerin was added to the final concentration of 1 nM. After 1 h incubation, supernatants were collected and chemerin concentration was assessed by ELISA. Chemerin concentration was normalized over the one of DMSO-treated control cells. The mean values of three independent experiments are shown. *p < 0.05; **p < 0.01; ***p < 0.001, by one-way ANOVA followed by Bonferroni’s multiple comparison test. **(C)** Hela cells stably expressing CCRL2 (CCRL2) or control cells (CTR) were incubated for 2 h in the absence (no chemerin) or in the presence of 1 nM chemerin. Cells were then washed with PBS (PBS), acidic buffer (pH3) or with acidic buffer followed by high salt buffer (pH3 + NaCl), for 5 min. Then, cells were lysed and whole cell lysates were analyzed by ELISA for chemerin concentration. The mean values of three independent experiments are shown. ***p < 0.001, by one-way ANOVA followed by Dunnett’s multiple comparison test. **(D)** Chemokine-like receptor 1 (CMKLR1)- or CCRL2-expessing HeLa cells or control cells were incubated with 1 nM chemerin for the indicated times. Supernatants were collected and chemerin concentration was assessed by ELISA. The values were normalized over no cells, at 1 h of incubation; the mean values of three independent experiments are shown. *p < 0.05 (CMKLR1- vs. CCRL2-expressing cells) by two-way ANOVA; CTR, control HeLa cells; CCRL2, HeLa cells stably expressing mCCRL2; CMKLR1, HeLa cells stably expressing mCMKLR1; no cells, supernatant incubated in the absence of cells; Dyn, Dynasore; ns, not significant.

C-C chemokine receptor-like 2 scavenging properties were analyzed in the past by several investigators yielding contrasting results (16, 23). To clarify this issue, HeLa cells stably expressing non-tagged CCRL2 were cultured in the presence of chemerin or CCL19. As shown in Figure 5A, a time-dependent decrease of chemerin, but not of CCL19, concentration was detected in the supernatant of CCRL2-expressing cells. As expected, no changes in chemerin or CCL19 concentrations were observed in the supernatants of non-transfected HeLa cells.

To elucidate whether the decrease in chemerin concentration in cell supernatants was due to the binding of the protein to the cell membrane and/or to CCRL2-mediated endocytosis, experiments were performed in the presence of dynasore, a dynamin inhibitor able to block endocytosis (42). Figure 5B shows that dynasore inhibited about 50% of chemerin consumption by CCRL2-transfected cells. These results suggest that, at least at the analyzed time point, only a fraction of chemerin is actively endocytosed while the rest is bound to the cell surface. Chemerin was quantified into the cell lysates obtained from chemerin-treated cells. Before lysis, cells were washed with either PBS alone, an acidic buffer, or an acidic buffer followed by a high salt buffer. Figure 5C shows that about 50% of cell-associated chemerin (found after incubation in PBS alone) could be removed from CCRL2-expressing cells by acidic and high salt washes. As expected, no chemerin was found associated with cells that do not express CCRL2. These results together with the data obtained in the presence of dynasore support the hypothesis that chemerin interacts with membrane CCRL2 and becomes internalized.

To further confirm these observations, HeLa cells stably expressing CMKLR1 were cultured in the presence of chemerin and its decrease from cell supernatants was analyzed. CMKLR1-positive cells removed chemerin from the supernatants in a time-dependent way (Figure 5D). Of note, chemerin consumption by CMKLR1-expressing cells was higher compared to the one of CCRL2-expressing cells (Figure 5D).

### CCRL2 Does Not Act As a Scavenger Receptor When Endogenously Expressed by Endothelial Cells

**Figure 6:**
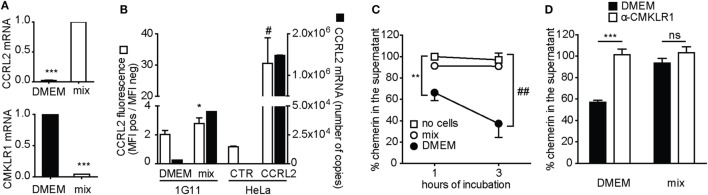
C-C chemokine receptor-like 2 (CCRL2) does not behave as a scavenger receptor when endogenously expressed by endothelial cells. 1G11 mouse endothelial cells were incubated overnight with medium (DMEM) or with IFNγ (50 ng/ml), TNFα (20 ng/ml), and LPS (1 µg/ml) (mix). **(A)** CCRL2 (above) or chemokine-like receptor 1 (CMKLR1) (below) mRNA expression in 1G11 mouse endothelial cells, at 18 h after treatment. 2^−ΔCT^ values were normalized over mix (for CCRL2 expression) or over DMEM (for CMKLR1 expression); the average values of four independent experiments are shown. **(B)** CCRL2 protein (white) and mRNA (black) expression in 1G11 cells, in control HeLa cells or in HeLa cells stably expressing CCRL2, evaluated by flow cytometry and qPCR, respectively. For protein expression, median values of fluorescence intensities of labeled cells were normalized over median of fluorescence intensities of unlabeled cells. For mRNA expression, the number of copies of CCRL2 is reported; quantification was performed using a plasmid standard curve. The mean values of four independent experiments are shown. **(C)** 18 h after incubation in the presence or in the absence of the mix of cytokines (mix), cells were incubated with 1 nM chemerin. At the indicated times, supernatants were collected and chemerin concentration was assessed by ELISA. Chemerin concentration was normalized over the one of no cells at 1 h; the mean values of three independent experiments are shown. **(D)** 1G11 Cells were pre-incubated with 0.1% DMSO or 10 µM mCMKLR1 antagonist for 10 min, then chemerin was added to the final concentration of 0.1 nM. After 1 h incubation, supernatants were collected and chemerin concentration was assessed by ELISA. Chemerin concentration in each sample was normalized over the concentration of chemerin incubated in the absence of cells (not shown in the graph); the mean values of three independent experiments are shown. ***p < 0.0001 by Student’s *t*-test; *p < 0.05 (mix vs. DMEM) and #*p* < 0.05 (CCRL2 vs. CTR), by Student’s *t*-test; **p < 0.01 and ##*p* < 0.001 by two-way ANOVA; ns, not significant. CTR, control HeLa; CCRL2, HeLa stably expressing mCCRL2; no cells, supernatant incubated in the absence of cells; mix, IFNγ + TNFα + LPS.

To investigate the scavenging properties of CCRL2 in a more physiological cell system, mouse blood endothelial cells (1G11) were used (15). In resting conditions, 1G11 cells express CMKLR1 but only very low levels of CCRL2 mRNAs. When 1G11 cells are stimulated with proinflammatory cytokines, the two receptors are modulated in an opposite manner with the upregulation of CCRL2 and the downregulation of CMKLR1 (Figure 6A). Of note, CCRL2 expression in cytokine-stimulated 1G11 cells is much lower than what observed in CCRL2-transfected HeLa cells, being about 40-fold lower (mRNA) and 10-fold lower (membrane protein), respectively (Figure 6B). When resting 1G11 cells were cultured in the presence of chemerin, a time-dependent reduction of chemerin concentration was observed in the cell supernatant. Conversely, culturing of chemerin-treated cytokine-activated 1G11 cells did not cause any change in chemerin levels (Figure 6C). Since resting 1G11 cells express high levels of CMKLR1, but also very low levels of CCRL2, the role of CMKLR1 in chemerin consumption was investigated. Figure 6D shows that treatment of 1G11 resting cells with a CMKLR1 selective antagonist (43) completely abrogated chemerin decrease in the supernatant. Conversely, the CMKLR1 antagonist did not show any effect when added to stimulated cells (Figure 6D).

## Discussion

This study was performed to investigate the internalization and scavenging properties of CCRL2, a 7-TMD receptor that shares structural similarities with the members of the ACKR family (44). Similar to ACKRs, CCRL2 does not activate G protein-dependent signaling or chemotaxis. However, the ability of CCRL2 to internalize and degrade its ligand still represents an open and controversial question (7, 23). Since it is possible that the discrepancies among different studies are due to technical artifacts, possibly related to the quality of commercially available moAbs, we decided to reevaluate CCRL2 internalization properties using a more direct approach that has been already used to successfully investigate intracellular trafficking of other 7-TMD receptors (27). For this purpose, CCRL2 was tagged at its extracellular N-terminus with a small peptide that can be covalently bound to a fluorophore by a highly specific enzymatic reaction (28). By using this technique, receptor labeling is chemically stable, is not sensitive to acidic pH and marks only receptors expressed at the cell membrane in living cells.

By this labeling approach, ectopically expressed CCRL2 was observed to undergo constitutive internalization and recycling to the plasma membrane following the slow recycling pathway. The analysis of CCRL2 internalization suggested that CCRL2 behaves differently from both ACKRs and classical chemotactic receptors. Indeed, the degree of CCRL2 internalization was lower than that observed for CXCR2 and CMKLR1, two signaling chemotactic receptors which, in turn, showed a very modest degree of internalization when compared to ACKR3, a well characterized scavenger receptor (1). In addition, the kinetics of CCRL2 internalization was not modified by treatment with chemerin or by several other chemokines. On the contrary, the internalization of CMKLR1, the chemerin functional receptor, was significantly increased following chemerin stimulation. Noteworthy, similar patterns of internalization were observed using two different recipient cells (HeLa and COS-7 cells), indicating that the cellular context in which the receptor is expressed does not modify CCRL2 trafficking. The finding that the C-terminus and the putative H8 are dispensable for CCRL2 internalization further differentiates CCRL2 from the ACKR family members (e.g., ACKR3) (27). H8 is a sequence predicted to interact with arrestins (40), a family of proteins that play an important role in clathrin-driven internalization (45). Therefore, the finding that H8 is dispensable for CCRL2 internalization is consistent with a recent report, showing that CCRL2 does not associate with β-arrestin1 and 2 (7). Although it should be noted that β-arrestin-independent internalization was reported to occur also with some chemokine receptors, such as CXCR3 (46).

Since one of the ACKR hallmarks is the ability to scavenge chemokines as a way to control inflammation (3), it was of interest to investigate whether the slow rate of CCRL2 internalization is associated with ligand scavenging. Our experiments demonstrate that CCRL2 promotes a time-dependent decrease of chemerin concentration in the supernatant of transfected cells. However, in the same experimental conditions, ectopic expression of CMKLR1 was associated with even a higher ability to remove chemerin from the supernatant. It is worth to consider that CMKLR1 is a GPCR that, similar to other chemotactic receptors, activates signaling and internalizes after ligand binding (7). This type of ligand internalization cannot be compared to the ligand scavenging ability that characterizes ACKRs. Therefore, chemerin endocytosis mediated by CMKLR1 and CCRL2 when ectopically expressed in transfected cells is an effect related to the biological behavior shared by all chemotactic receptors (47) and does not reflect the specialized scavenging functions displayed by ACKRs.

To further investigate CCRL2 scavenging properties, a more physiological cell system was used, namely primary cultures of mouse endothelial cells (15, 26). Resting 1G11 endothelial cells induced a CMKLR1-dependent decrease of chemerin concentration in the cell supernatant and this effect was fully blocked by a CMKLR1 antagonist. On the contrary, no change in chemerin concentration was observed in the presence of activated endothelial cells. Activated 1G11 cells are known to upregulate CCRL2 and simultaneously downregulate CMKLR1 expression. The apparent discrepancy between the results obtained with CCRL2-stably transfected HeLa cells and activated endothelial cells is likely to reside in the 40-fold higher expression of CCRL2 in transfected cells compared to the physiological expression observed in activated endothelial cells. Ultimately, these data fully support the proposed function of CCRL2 as a membrane-anchoring protein for the presentation of chemerin to CMKLR1-expressing cells (16, 19).

In conclusion, this study demonstrates that CCRL2 does not possess two of the hallmarks of ACKRs: first, the ability to undergo rapid, constitutive, membrane internalization; second, the ability to scavenge the ligand from the cell supernatant. These aspects clearly differentiate CCRL2 from the other members of the ACKR family. By sequence homology with chemokine receptors, CCRL2 has always been cited as a receptor. However, a hallmark and prerequisite for defining a molecule as a receptor is the ability to trigger intracellular signaling. Thus, without evidence of such a signaling, at the moment, CCRL2 should be classified simply as a chemerin binding protein (48). Nonetheless, the data obtained using CCRL2 KO mice clearly support a crucial role of CCRL2 in the regulation of the immune response *in vivo* (10, 13, 15, 19, 20). Further studies are required to fully elucidate the mechanisms by which CCRL2 regulates immune functions.

## Author Contributions

CM, VG, and LT performed and analyzed the experiments. DB, AP, and MT contributed to the organization and interpretation of the experiments and reviewing of the manuscript. SS planned the experiments, directed the entire project, and together with CM wrote the manuscript.

## Conflict of Interest Statement

The authors declare that the research was conducted in the absence of any commercial or financial relationships that could be construed as a potential conflict of interest.

## Abbreviations

CCRL2, C-C chemokine receptor-like 2; ACKR, atypical chemokine receptor; 7-TMD, seven-transmembrane domain; GPCR, G protein-coupled receptor; CMKLR1; chemokine-like receptor 1; ACP, acyl carrier protein; CXCR2, C-X-C motif chemokine receptor 2; EEA1, early endosome antigen 1; TfR, transferrin receptor; LAMP1, lysosomal-associated membrane protein 1; VAMP2, vesicle-associated membrane protein 2; VAMP3, vesicle-associated membrane protein 3; H8, helix-VIII; ELISA, enzyme-linked immunosorbent assay.
