# Supplementary material for: The Atypical Receptor CCRL2 (C-C Chemokine Receptor-Like 2) Does Not Act As a Decoy Receptor in Endothelial Cells
Source: Front Immunol. 2017 Oct 6;8:1233. doi: 10.3389/fimmu.2017.01233 (PMC5635198; doi:10.3389/fimmu.2017.01233)
Supplement: Supplementary file 2 [file Supplementary_File_1.PDF]

Supplementary Material

C-C chemokine receptor like 2 (CCRL2) is not a scavenger receptor

Chiara Mazzotti, Vincenzo Gagliostro, Daniela Bosisio, Annalisa del Prete, Laura Tiberio, Marcus Thelen, Silvano Sozzani*

*** Correspondence:** Prof. Silvano Sozzani silvano.sozzani@unibs.it

1 Supplementary Viedos

Supplementary Video 1. Membrane recycling of CCRL2$^+$ vesicles. COS-7 cells were transfected with ACP-CCRL2 and membrane CCRL2 was labelled. Cells were placed under the microscope at 37°C, in the presence of 5% CO2, and observed by time-lapse microscopy every 5 seconds. Blue line, endocytosing vesicle; green line, exocytosing vesicle.

2 Supplementary Figures

Supplementary Figure 1

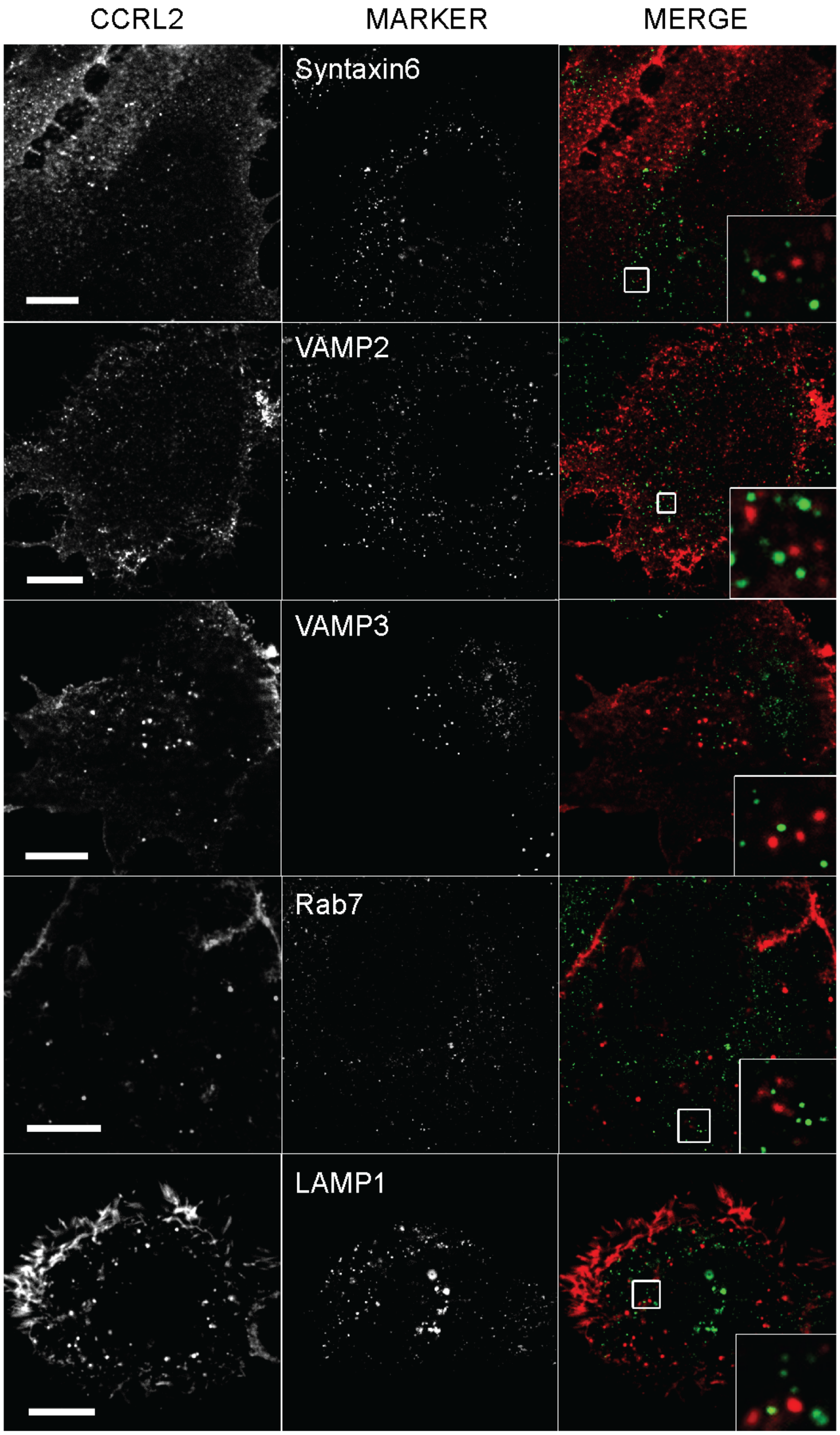

Supplementary Figure 1. CCRL2 fluorescent vesicles do not co-localize with markers of lysosomes and Golgi. COS-7 cells were transiently transfected with ACP-CCRL2 and membrane

CCRL2 was labelled. Cells were then placed at 37°C for 20 min, fixed, permeabilized and mounted after staining for the indicated markers. Images were taken at 100x magnification. Labelled CCRL2 and the intracellular markers are shown in the first and the second columns, respectively. The third column represents the merging of CCRL2 (red) and marker (green) fluorescences. Inserts represent magnifications of the boxed areas. Scale bars: 10 µm.
